# Evaluation of the Quality and Readability of Web-Based Information Regarding Foreign Bodies of the Ear, Nose, and Throat: Qualitative Content Analysis

**DOI:** 10.2196/55535

**Published:** 2024-08-15

**Authors:** Tsz Ki Ko, Denise Jia Yun Tan, Ka Siu Fan

**Affiliations:** 1 Department of Surgery Royal Stoke Hospital Stoke United Kingdom; 2 Department of Surgery Royal Surrey County Hospital Guildford United Kingdom

**Keywords:** foreign body, quality of internet information, readability of internet information, EQIP, Ensuring Quality Information for Patients, medical informatics, readability, readable, health information, online information, information resource, information resources, website, websites, quality, evaluation, evaluations, reading level, grade level

## Abstract

**Background:**

Foreign body (FB) inhalation, ingestion, and insertion account for 11% of emergency admissions for ear, nose, and throat conditions. Children are disproportionately affected, and urgent intervention may be needed to maintain airway patency and prevent blood vessel occlusion. High-quality, readable online information could help reduce poor outcomes from FBs.

**Objective:**

We aim to evaluate the quality and readability of available online health information relating to FBs.

**Methods:**

In total, 6 search phrases were queried using the Google search engine. For each search term, the first 30 results were captured. Websites in the English language and displaying health information were included. The provider and country of origin were recorded. The modified 36-item Ensuring Quality Information for Patients tool was used to assess information quality. Readability was assessed using a combination of tools: Flesch Reading Ease score, Flesch-Kincaid Grade Level, Gunning-Fog Index, and Simple Measure of Gobbledygook.

**Results:**

After the removal of duplicates, 73 websites were assessed, with the majority originating from the United States (n=46, 63%). Overall, the quality of the content was of moderate quality, with a median Ensuring Quality Information for Patients score of 21 (IQR 18-25, maximum 29) out of a maximum possible score of 36. Precautionary measures were not mentioned on 41% (n=30) of websites and 30% (n=22) did not identify disk batteries as a risky FB. Red flags necessitating urgent care were identified on 95% (n=69) of websites, with 89% (n=65) advising patients to seek medical attention and 38% (n=28) advising on safe FB removal. Readability scores (Flesch Reading Ease score=12.4, Flesch-Kincaid Grade Level=6.2, Gunning-Fog Index=6.5, and Simple Measure of Gobbledygook=5.9 years) showed most websites (56%) were below the recommended sixth-grade level.

**Conclusions:**

The current quality and readability of information regarding FBs is inadequate. More than half of the websites were above the recommended sixth-grade reading level, and important information regarding high-risk FBs such as disk batteries and magnets was frequently excluded. Strategies should be developed to improve access to high-quality information that informs patients and parents about risks and when to seek medical help. Strategies to promote high-quality websites in search results also have the potential to improve outcomes.

## Introduction

Foreign bodies (FBs) in the upper respiratory and digestive tract account for approximately 11% of admissions to ear, nose, and throat (ENT) emergency services [1]. While most FB can be removed by emergency clinical staff on initial presentation [2], challenging cases may require removal under general anesthesia, particularly if the FB is lodged or impacted within the aerodigestive tract, nasal cavity, or ear canal [3]. While this is thought to primarily affect children [4], it also causes significant problems in adult populations [2]. For example, a recent systematic review of FBs identified that adults represent a small but significant proportion of FB cases presenting to emergency services [5]. Regardless of age, it is recommended that the identification of FBs, of both organic and inorganic nature, be treated with a high degree of clinical suspicion to ensure safe and effective care [5].

With FB inhalation or insertion affecting children, much of its safety advice is aimed at educating the adults responsible to increase awareness and reduce risks and complications [6,7]. One of the primary goals would be to raise public awareness of recognizing FBs within the aerodigestive tract [8]. As the speed of response may be critical in such an event, educating the public on what to do is also a priority [8,9]. This approach to providing safety advice is underpinned by the principles of the health belief model [10], whereby important health-related actions are more likely to be taken when individuals are aware of both the benefits and steps of the action as well as the risks [11]. The quality of FB-related safety advice is therefore central to this process [12].

With FBs often presenting urgently, a layperson may turn to information on the internet for immediate advice. As with other common conditions, a plethora of websites provide information and advice on the insertion, ingestion, or inhalation of FBs; the quality of these websites may vary considerably, as demonstrated in other ENT studies [13,14]. The impact of information can be optimized if the content is readable, accurate, and easily comprehended by individuals [15].

This study aimed to assess the quality and readability of online safety advice regarding FB insertion, ingestion, and inhalation. A variety of tools have been developed to assess the quality and readability of written information, so this study uses validated tools that have commonly been used in similar studies previously to allow greater comparability of results. For assessment of quality, the Ensuring Quality Information for Patients (EQIP) tool is a validated tool to assess written material quality, designed specifically for health professionals and researchers [16]. The Flesch Reading Ease score (FRES) [17], Flesch-Kincaid Grade Level (FKGL) [18], Gunning-Fog Index (GFI) [19], and Simple Measure of Gobbledygook (SMOG) [18] are commonly used readability scores, which use different methods to estimate the literacy levels required to adequately understand the text.

## Methods

### Study Design

Google, the most widely used English language search engine, was used to identify websites [20]. Only Google was used as previous studies returned similar results when multiple search engines were used [21]. As it is not possible to capture all possible search terms used by the public, Google Trends was used to assess and select the 6 most popular search terms based on their relative popularities: “object in ear,” “object in the nose,” “object in the throat,” “ear foreign body,” “nose foreign body,” and “throat foreign body.” Only the first 30 results were captured for each search term as most users do not view beyond the first page [22]. As the Google search location setting affects results presented, searches were carried out with the country set as Australia, Canada, the United Kingdom, and the United States, which are the countries with the highest number of native English speakers. This does not restrict results to websites from other countries from being displayed and instead provides results representative of what English-speaking users would search and find.

### Eligibility and Assessment

Eligibility criteria are listed in Textbox 1. All websites providing health information or advice regarding FBs or objects in the ear, nose, or throat were included. Health information was included if it was in a written format and intended for the general public or patient demographic. Websites that were primarily video based or locked behind paywalls were excluded. Non-English language websites were excluded because this study intended to assess the information available to English speakers and because the 2 researchers were not capable of assessing quality and readability in other languages. Websites intended for health care professionals and academic journal papers were also excluded. Duplicate hyperlinks were removed before the assessment. Websites were assessed independently by 2 authors (TKK and DJYT) per our previous ENT-focused EQIP study [23]. The senior author (KSF) provided supervision of assessments and input for any scoring discrepancies.

**Eligibility criteria.**

**Inclusion criteria**

- Written health information
- Health information relating to foreign bodies
- Written in the English language

**Exclusion criteria**

- Video-based information
- Information that mentions foreign bodies but is focused on a different health or other issue
- Content aimed at health care professionals or academics
- Non-English content

### Modified EQIP Tool

The 36-item EQIP tool was used to evaluate each website. This is widely considered to be a robust means of examining both the content and the design of health-related advice [15], as well as considering the needs of caregivers or parents within this process [15]. To assess the quality of content, the use of EQIP will therefore seek to highlight the current quality of safety advice regarding FBs at this time. For each item, a “yes,” “no,” or “not applicable” contributed to 1 or 0 points. The EQIP tool is comprised of 3 domains: content, identification, and structure. The content domain provided information on the main topic and the relevant management (items 1-18). The identification domain evaluates the production details, including authorship, publication date, and bibliography (items 19-24). The structure domain (items 25-36) examines website usability and overall presentation.

### Readability Assessment

Apart from EQIP’s structure domain, the readability of each website may be assessed using validated scoring systems: FRES, FKGL, and GFI. Following previous literature, these scores were calculated using an online tool [24]. Only the plain text in the article content was assessed, without figures, images, or legends. The calculated results included the number of education years required for correlating literacy level [24]. FRES uses the average sentence length in words to assess its readability, calculated by *206.835 –  (1.015 × total words / total sentences) – (84.6 × total syllables / total words)*. This average number of words with higher scores represents easier readability [17]. FKGL is developed for the same purpose but uses the number of syllables in its sentences, calculated by *(0.39 × total words / total sentences) + (11.8 × total syllables / total words) – 15.59*. GFI similarly assesses readability that focuses on complexity and factors in words with 3 or more syllables [15]. This is calculated as *(0.4 × words / sentences) + (100 × complex words / words)*. As FKGL and GFI both correspond to the reading level with the amount of education, it is interpreted inversely: higher scores will indicate higher difficulty or literacy required [19]. Moreover, this study included the use of the SMOG readability score, which focuses on polysyllabic words alone [18]. The lack of a gold standard in assessing readability meant that this set of readability tools was validated for a comprehensive assessment and known to be widely applicable across different domains [16].

### Data Collection and Additional Assessments

Website demographic details were recorded, including the country and type of source. Content from nongovernmental organizations that oversee public health was classified as “charity or nongovernmental organizations.” Other nonprofit groups included those that were patient led (“patient group”) and professional led (“professional society”). All for-profit organizations were classified as “industry or private.”

Any acute advice or FB removal methods were recorded in detail. The discussion of specific FBs, and morbidity or mortality rates was recorded. Any disclaimers on websites to seek formal assessment by a health care professional were also noted.

### Statistical Analysis

We used SPSS (version 25; IBM Corp) software data analysis to analyze data. The descriptive values in the form of mean, median, IQR, and aggregated scores were demonstrated where applicable. EQIP and readability scores were analyzed with a 1-way ANOVA between subgroups. An α level of *P*<.05 was considered statistically significant.

### Patient and Public Involvement

There was no patient or public involvement in the conception, design, or data collection of the study or the production of the manuscript.

### Search Outcome

**Figure 1:**
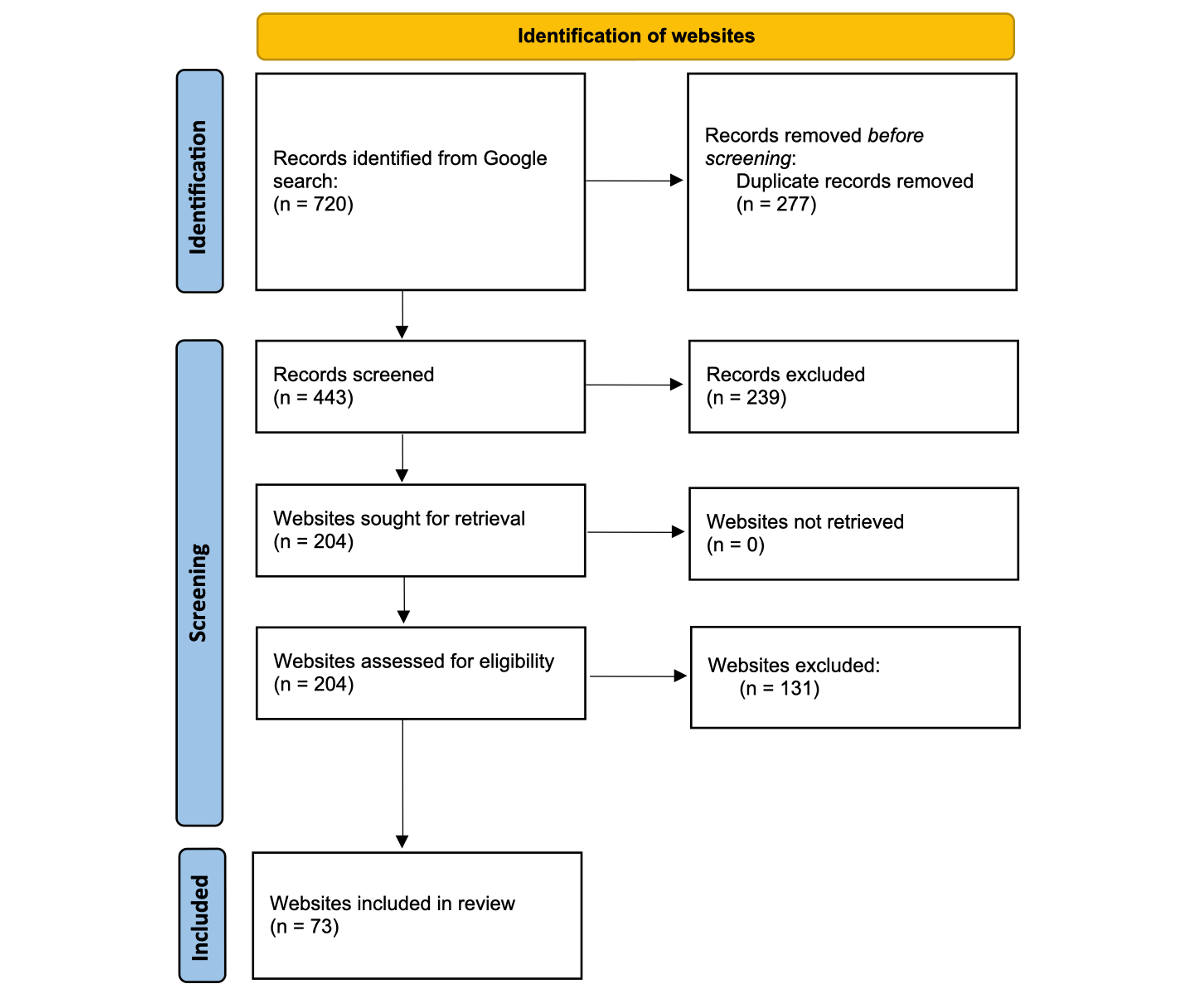
Flow diagram illustrating the stages of the website search process. Records were excluded during screening due to not being focused on FBs (n=210), use of video or images with no text (n=20), and being aimed at an academic or professional, rather than a general audience (n=19).

In total, 720 websites were identified. Further, 73 websites remained for analysis after the removal of duplicates and websites that did not meet inclusion criteria. The PRISMA (Preferred Reporting Items for Systematic Reviews and Meta-Analyses) flowchart for the inclusion of websites qualifying for analysis is shown in Figure 1.

## Results

### EQIP Performance and Demographics

The breakdown of individual EQIP performance is illustrated in Multimedia Appendix 1. The overall median EQIP score was 21 (IQR 18-25; range 11-29). General EQIP data property is shown in Table 1. The 75th percentile of the total EQIP score is 25 or above, achieved by 18 websites. These websites were considered high scoring.

**Table 1:** The median, IQR, and range of the scores for the websites included are shown disaggregated for the content, identification, and structure domains of EQIP^a^. The IQR shows considerable variation in the overall EQIP as well as the content domain.

Score	Content	Identification	Structure	Overall EQIP
Median (IQR)	11 (9-13)	3 (2-4)	8 (7-8)	21 (18-25)
Range	3-15	0-6	4-10	11-29
99th percentile	15	5	10	29

^a^EQIP: Ensuring Quality Information for Patients.

In total, 63% (n=46) of the 73 websites originated from the United States, with a median EQIP score of 21 (IQR 18.25-24). This was followed by Australian (n=10, 14%; median EQIP score 21.5, IQR 19.25-27) and Canadian (n=9, 12%; median EQIP score 23, IQR 20-25) websites. Australia-based websites (n=10) had the widest IQR of 7.75. Further, 1 (1%) website originated from India, with an EQIP score of 26.

The most common source of information was industry (n=21, 29%) and government or health departments (n=15, 20%), with median EQIP scores of 21 (IQR 17-25) and 23 (IQR 20-27), respectively. Charity or nongovernment organizations (n=2, 3%), on the other hand, had the lowest median EQIP score of 17.5 (IQR 16.75-18.25; Table 2).

**Table 2:** Descriptive analysis of websites grouped by country of origin and source of information.

Variable			Websites (n=73), n (%)		EQIP^a^ score, median (IQR)
**Country**					
	India	1 (1)		26.0 (N/A^b^)	
	Canada	9 (12)		23.0 (20-25)	
	Australia	10 (14)		21.5 (19.25-27)	
	United States	46 (63)		21.0 (18.25-24)	
	United Kingdom	7 (10)		19.0 (17-24)	
**Source of information**					
	Professional society (nonprofit groups of health care professionals)	11 (15)		24 (20-26)	
	Government or health department	15 (20)		23 (20-27)	
	Patient group (primarily serve patients [25])	1 (1)		23 (23-23)	
	Hospital (any organization that provides hospital care)	11 (15)		22 (18.5-23.5)	
	Industry (for-profit organization within the medical industry, including clinics)	21 (29)		21 (17-25)	
	News service (both primary and secondary news websites that are not written for professionals)	2 (3)		20 (N/A)	
	Academic center (academic institutions)	10 (14)		19.5 (16.75-20.75)	
	Charity or nongovernmental organization (oversee a broader demographic, like the Red Cross and WHO^c^)	2 (3)		17.5 (16.75-18.25)	

^a^EQIP: Ensuring Quality Information for Patients.

^b^N/A: not applicable.

^c^WHO: World Health Organization.

### EQIP: Content Data

Overall content data median EQIP score was 11 (61%) out of 18, with a maximum score of 15 (83%). All high-scoring websites met the requirements for items 1, 2, 3, 4, 7, and 9. Most websites (69/73, 95%) also mentioned alert signs that the patient may detect (item 14) and provided coverage of all relevant issues for the topic (item 18). However, many websites (68/73, 93%) failed to address the medical intervention costs and insurance issues (item 15).

### EQIP: Identification

The overall median score for the identification section obtained was 3 (50%) out of 6, with a maximum score of 6 (100%; Table 1). Over 70% of the 73 websites included items 19 (n=53, 73%), 20 (n=65, 89%), and 21 (n=54, 74%). However, almost all websites (n=72, 99%) failed to fulfill item 24. The only website that included item 24 had the highest EQIP of 29, achieved by Healthdirect from Australia. While there is no significant difference in overall EQIP score between different sources of information (*P*=.20), the EQIP scores specifically for the identification domain showed significant differences (*P*=.001).

### EQIP: Structure

The median score obtained for the structure domain was 8 (66%) out of 12, and the highest score obtained was 10 (83%). All websites included items 25, 27, 28, 29, 30, 32, and 33. Further, at least 52% of the 73 websites failed to achieve items 31 (n=38, 52%) and 35 (n=66, 90%).

### Top EQIP Scoring Websites

Only 3 websites (median EQIP score 29) fulfilled the EQIP cutoff score for the 99th percentile. In total, 2 of these were produced by Healthdirect, a government website based in Australia, and the Royal College of Emergency Medicine Learning, a professional society website based in the United Kingdom.

### Readability Assessment

**Figure 2:**
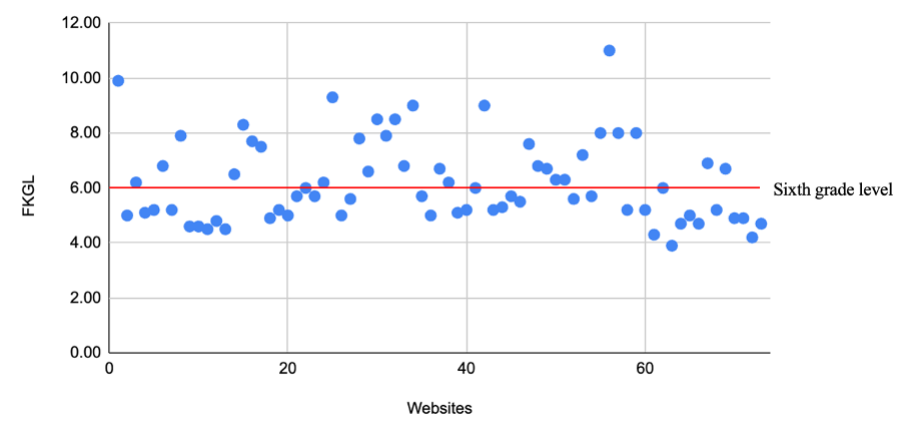
Scatterplot analysis of FKGL scores; only 3 websites are at the recommended reading level (red line). FKGL: Flesch-Kincaid Grade Level.

Overall, the websites’ mean FRES and FKGL scores were 63.9 (SD 10.3; range 37.6-81.7) and 6.2 (SD 1.5; range 3.90-14.0), respectively. These scores reflect a mean reading age of 12.4 (SD 1.3; range 10.0-18.0) years. Further, 41 (56%) websites achieved the recommended sixth grade or below readability level (Figure 2). The FRES did not have statistically significant differences between countries (*P*=.62).

Readability scores demonstrated statistically significant differences across sources of information: the GFI (*P*=.02) and SMOG (*P*=.03). In addition, the Pearson correlation showed no correlation between overall EQIP scores and the readability of website content.

### Additional Information

In total, 95% (n=69) of the 73 websites mentioned the different types of FB, with disk battery being the most mentioned (n=51, 67%), followed by toys (n=30, 41%); peas, beans, or nuts (n=28, 38%), insect (n=28, 38%); and bead (n=25, 34%). Only 14 (19%) websites mentioned magnets. Different removal methods have been discussed by 82% (n=60) of the websites. FB removal by instruments was most mentioned (n=44, 60%), whereas endoscopic removal was least mentioned (n=9, 12%). Most websites 89% (n=65) provided patients with acute advice by advising them to seek immediate medical attention. Further, 38% (n=28) advised patients to try and remove FB by themselves if safe to do so. Fewer than 10% of websites included first-aid measures, such as backslaps, encouraging coughing, and the Heimlich maneuver. Only 1 (1%) website discussed the mortality rate of FB and 6 (8%) websites mentioned overall complication rates.

## Discussion

### Principal Findings

This was the first study to evaluate web-based information available to patients regarding FBs in the aerodigestive tract using validated tools like EQIP and various readability assessments [26]. The main finding suggests that the available information is of only moderate quality, with a median EQIP score of 21 (IQR 18-25; maximum 29). This is significantly lower than the maximum possible EQIP score of 36 [27]. The IQR of 7 (median EQIP 21) indicates high variability in the quality of easily accessible FB information. The readability scores (FKGL 6.2, GFI 6.5, SMOG 5.9, and FRES 12.4 years) suggested that the information was generally at the recommended 6th-grade reading level.

### Evaluation of Quality of Health Information

Although this is the first study on FBs, previous studies investigating the quality of information on ENT conditions have also found web-based information to be of moderate or poor quality. A study of 295 websites offering information on rhinoplasty conducted by Shamil et al [28] found a median EQIP score of only 17, which the authors noted may lead to unrealistic expectations among patients about the potential outcomes of rhinoplasty. A more recent study by the same group found a median EQIP score of 20 when investigating the quality of web-based information relating to cosmetic injectable fillers [14]. The authors found that although the websites included (n=172) did provide some information on the risks of fillers, the majority of websites failed to disclose major risks. It may be that the EQIP scores for information regarding elective, cosmetic procedures are lower than this study (median EQIP score 21) due to the urgent nature of FBs and the desire to present information that could help with rapid decision-making.

In contrast to these studies, which investigated common elective procedures, the urgent nature of FBs means that financial considerations are unlikely to influence the presentation of information. Furthermore, it appears that commercial interests are not the key driver of a reduction in information quality: similarly poor content quality was seen in other infective conditions that affect airways, such as tonsillitis (median EQIP score 19) [13] and COVID (median EQIP score 17.8) [29]. This suggests that moderate and poor-quality information is likely the norm for ENT-related complaints regardless of its commercial influences.

To analyze the quality of information relating to FBs in greater detail, the inclusion of specific guidance was also considered. Precautionary measures are an important aspect of FB guidance because they can prevent the occurrence of FB events in the first place [30]. For example, information about age-appropriate toys for young children can help to prevent ingestion, insertion, and inhalation of small parts [31]. However, only 59% (43/73) of the websites included in this study contained any information on precautionary measures, suggesting this is a potential area for improvement and greater awareness. Clear advice about keeping small, ingestible objects out of reach of children and buying age-appropriate toys should be a priority for FB information [30].

Certain FBs, such as peas, seeds, magnets, and disk batteries, are particularly common [32]. Disk batteries specifically present a serious risk as the short distance between both faces of the battery can lead to electrical circuits being formed through tissue, leading to burns, perforations, and fistulations. It is recommended that button batteries lodged in the esophagus are removed immediately, although they pose less of a threat when they enter the stomach due to the reduced risk of circuit formation with tissues [33]. Given its importance, this is inadequately reflected in the website cohort as only 70% (51/73) mentioned it. Alternatively, peas are commonly inserted and were only mentioned in 38% (28/73) of websites. If peas are not removed promptly from the nose, they can swell [34] which most websites failed to mention. Similarly, magnets are known to cause pressure necrosis but were only mentioned by 19% (14/73) of websites included in this study.

This highly varied discussion in risky FBs suggests that items perceived to be particularly hazardous (eg, batteries) receive more focus than “safer” items like peas, seeds, and magnets. Without adequate emphasis on the high level of hazard to health, this may mislead patients or caregivers about potentially serious FB insertion. As well as the low prevalence of some risk factors in the web-based literature, there was also a lack of description of the complications caused by these FBs and the management of these complications if they occur. Management approaches for complications were also absent in the majority (46/73, 63%) of websites. Information should differentiate between cases where x-rays are suggested, such as disk battery or magnet ingestion, and those cases where the child is asymptomatic or has ingested a radio-translucent object [35].

When considering the advice and suggestions to resolve an FB event, websites tended to avoid advising self-management in favor of seeking professional medical assistance. Basic first aid measures such as backslaps, encouraging coughing, and the Heimlich maneuver [31] were mentioned in fewer than 10% of websites, while the vast majority (n=65, 89%) advised patients to seek immediate medical attention. This provision of caution advice may be useful in less acute presentations but, in cases of emergencies, having easily accessible first-aid information may save lives while awaiting medical attention to arrive.

As well as limited advice about first aid, only 38% (n=28) of websites advised patients to try to remove FBs themselves. Where discussed, this was stipulated to only do so when deemed safe. Part of the reason for this low figure may be due to difficulties in conveying when self-management is deemed safe [30] and that the information provider does not want to be held liable for the consequences of failed attempts. A standardized guideline or checklist produced by ENT specialists could be a way to provide reliable information on this subject. This could be released with an open license and sent directly to those websites that appear in the top Google results for common search terms. Another issue raised by the present research is whether the top Google search results reflect the highest-quality information available. As well as improving the quality of information, there is the potential for future research and practice to focus on adjusting search engine algorithms to promote better quality health information to higher positions in the results using artificial intelligence technologies.

Notably, the majority of websites (69/73, 95%) provided information identifying “red flags” in a problematic FB event. This appropriately reflects the urgent nature of the emergency and at least provides the public with some interim advice while awaiting the attendance of a trained health care professional. Similarly, for incidents that are not immediately life-threatening, providing adequate information may assist with the self-triage process [36]. However, 52% (38/73) of websites failed to provide balanced information about the benefits and risks of interventions to remove or treat FBs, which could lead to patients inappropriately seeking medical intervention with potential iatrogenic complications, or failing to seek intervention when it is recommended.

Encyclopedias, like Wikipedia, are often highly ranked across search engine results but, these were not included in our search. The most relevant Wikipedia article, “Foreign Body Aspiration” [37]*,* contains useful information but its technical terminologies may be poorly aligned with the search terms used in this study. Research has shown [38] that Wikipedia can be a good source of health information if there is a concerted effort by those with sufficient knowledge and ability to edit the contents. However, patients should treat freely editable websites with extra caution as laypersons will not be able to distinguish between high and low-quality Wikipedia entries. There does not appear to be any previous literature that has investigated EQIP scores for Wikipedia articles providing ENT information, but in studies into other health information, Wikipedia articles have performed similarly in terms of EQIP scores to other sources [39].

### Evaluation of Readability of Health Information

Overall, the websites were of an appropriate literacy level to accommodate the varied literacy levels of the public. Further, 3 of the readability measures (FRES, FKGL, and GFI) accounted for the average sentence length, the number of words, and the number of syllables per word to calculate a numerical measure of readability. Based on a recommended readability level of 11-12 years (sixth grade) for health literature [15], the majority of websites (41/73, 56%) were at or below this level but the mean FRES score was above it (12.4, SD 1.3 years), with an average FKGL of 6.2 (SD 1.5). While the mean FRES was slightly above target, this can represent a generally acceptable range of readability of this content. Additionally, there was no correlation between readability and quality scores, suggesting that both high- and low-quality content were being produced at a generally acceptable readability level. This is comparable with previous studies which have shown a poor correlation between EQIP and readability measures [29].

### Limitations and Further Recommendations

Several limitations should be considered when interpreting this study. First, this study was limited in scope to English language results and might not be representative of all web-based advice on FBs worldwide. Translational tools were avoided as their accuracy may influence the flow, content, and readability of content in other languages which would misrepresent the original content. Similarly, many eligible web pages were not captured within the first 3 pages of results, but this maintains validity as patients are unlikely to read beyond. The results also only represent a cross-sectional assessment as results are subject to change and are tailored to individual users’ locations and search histories [40]. These tools were chosen as they were validated but their generalizability may still be affected by the subjective interpretation of assessors [41].

The FRES tool has been criticized for its reliance on the length of words as a means of calculating readability [42]. When considering health-related literature, there may be long words that would be well-known to patients researching their condition, such as neuroblastoma, which raises the FRES score even though they do not impair readability. Therefore, this study uses a combination of metrics to draw conclusions based on generalized readability scores, taking into account the number of sentences, words, and syllables.

Any future research and practice should focus on ways to improve the quality and readability of web-based information relating to FB insertion, ingestion, and inhalation. It would be beneficial to produce guidance for those writing web-based information containing the key risks posed by FBs and providing clear guidance about when FB removal at home is safe and when further medical advice should be sought. To produce this guidance, an expert panel of ENT specialists should be consulted. The aim of this guidance should be to increase understanding in the community of how to recognize and deal with problematic FBs. There is potential to improve the readability of the available web-based information using artificial intelligence technologies such as ChatGPT, which can write and rewrite the information in a variety of registers including simple English.

### Conclusions

This was the first study to investigate the quality and readability of web-based information about FB insertion, ingestion, and inhalation using validated tools (EQIP and FRES). The main conclusions of this study were that most websites were of inadequate quality but generally had acceptable readability scores for public use. To improve the timely presentation and management of FBs, the quality and readability of information available online should be improved to enable patients or family members to understand the risks presented by FBs and when to take action. In particular, information should be provided about red flag FBs such as disk batteries in all cases, and precautionary measures to prevent FB events. Future research should focus on ways in which FB health information which ranks highly on internet searches can be made high-quality and readable, such as through the dissemination of a standardized information pack produced by ENT professionals.

## Appendix

Multimedia Appendix 1 Aggregated EQIP performance breakdown of websites. EQIP: Ensuring Quality Information for Patients.

## Abbreviations

ENT: ear, nose, and throat
EQIP: Ensuring Quality Information for Patients
FB: foreign body
FKGL: Flesch-Kincaid Grade Level
FRES: Flesch Reading Ease score
GFI: Gunning-Fog Index
PRISMA: Preferred Reporting Items for Systematic Reviews and Meta-Analyses
SMOG: Simple Measure of Gobbledygook
