# Supplementary material for: Evaluation of the Quality and Readability of Web-Based Information Regarding Foreign Bodies of the Ear, Nose, and Throat: Qualitative Content Analysis
Source: JMIR Form Res. 2024 Aug 15;8:e55535. doi: 10.2196/55535 (PMC11362703; doi:10.2196/55535)
Supplement: Multimedia Appendix 1 [file formative_v8i1e55535_app1.docx]

**Multimedia Appendix 1.** Aggregated EQIP performance breakdown of websites. EQIP: Ensuring Quality Information for Patients.

| **Item** | **Yes (n, %)** | **No (n, %)** | **N/A (n, %)** |
| --- | --- | --- | --- |
| Content Data [1. Initial definition of which subjects will be covered] | 71 (97%) | 2 (3%) | 0 (0%) |
| Content Data [2. Coverage of the previously defined subjects (NA if the answer is “no” for item 1)] | 71 (97%) | 0 (0%) | 2 (3%) |
| Content Data [3. Description of the medical problem/treatment/procedure] | 68 (93%) | 5 (7%) | 0 (0%) |
| Content Data [4. Definition of the purpose of the interventions] | 60 (82%) | 13 (18%) | 0 (0%) |
| Content Data [5. Description of treatment alternatives (conservative management)] | 54 (74%) | 19 (26%) | 0 (0%) |
| Content Data [6. Description of the sequence of the interventions and surgical procedure] | 45 (62%) | 28 (38%) | 0 (0%) |
| Content Data [7. c] | 46 (63%) | 27 (37%) | 0 (0%) |
| Content Data [8. Description of the quantitative benefits to the patient] | 20 (27%) | 53 (73%) | 0 (0%) |
| Content Data [9. Description of the qualitative risks and complications] | 59 (81%) | 14 (19%) | 0 (0%) |
| Content Data [10. Description of the quantitative risks and complications] | 35 (48%) | 38 (52%) | 0 (0%) |
| Content Data [11. Addressing quality-of-life issues] | 21 (29%) | 52 (71%) | 0 (0%) |
| Content Data [12. Description of how complications are handled] | 27 (37%) | 46 (63%) | 0 (0%) |
| Content Data [13. Description of the precautions that the patient may take] | 43 (59%) | 30 (41%) | 0 (0%) |
| Content Data [14. Mention of alert signs that the patient may detect] | 69 (95%) | 4 (5%) | 0 (0%) |
| Content Data [15. Addressing medical intervention costs and insurance issues] | 5 (7%) | 68 (93%) | 0 (0%) |
| Content Data [16. Specific contact details for hospital services (NA if not hospitals)] | 7 (10%) | 4 (5%) | 62 (85%) |
| Content Data [17. Specific details of other sources of reliable information/support] | 41 (56%) | 32 (44%) | 0 (0%) |
| Content Data [18. Coverage of all relevant issues for the topic (summary item for all content criteria)] | 39 (53%) | 34 (47%) | 0 (0%) |
| Identification [19. Date of issue or revision] | 53 (73%) | 20 (27%) | 0 (0%) |
| Identification [20. Logo of the issuing body] | 65 (89%) | 8 (11%) | 0 (0%) |
| Identification [21. Names of the persons or entities that produced the document] | 54 (74%) | 19 (26%) | 0 (0%) |
| Identification [22. Names of the persons or entities that financed the document] | 8 (11%) | 65 (89%) | 0 (0%) |
| Identification [23. Short bibliography of the evidence-based data used in the document] | 27 (37%) | 46 (63%) | 0 (0%) |
| Identification [24. Statement about whether and how patients were involved/consulted in the document's production] | 1 (1%) | 72 (99%) | 0 (0%) |
| Structure data [25. Use of everyday language and explanation of complex words or jargon] | 72 (99%) | 1 (1%) | 0 (0%) |
| Structure data [26. Use of generic names for all medications or products (NA if no medications described)] | 6 (8%) | 1 (1%) | 66 (90%) |
| Structure data [27. Use of short sentences (<15 words on average)] | 73 (100%) | 0 (0%) | 0 (0%) |
| Structure data [28. Personal address to the reader] | 63 (86%) | 10 (14%) | 0 (0%) |
| Structure data [29. Respectful tone] | 73 (100%) | 0 (0%) | 0 (0%) |
| Structure data [30. Clear information (no ambiguities or contradictions)] | 71 (97%) | 2 (3%) | 0 (0%) |
| Structure data [31. Balanced information on risks and benefits] | 35 (48%) | 38 (52%) | 0 (0%) |
| Structure data [32. Presentation of information in a logical order] | 72 (99%) | 1 (1%) | 0 (0%) |
| Structure data [33. Satisfactory design and layout (excluding figures or graphs; see next item)] | 70 (96%) | 3 (4%) | 0 (0%) |
| Structure data [34. Clear and relevant figures or graphs (NA if absent)] | 13 (18%) | 0 (0%) | 60 (82%) |
| Structure data [35. Inclusion of a named space for the reader's notes or questions] | 7 (10%) | 66 (90%) | 0 (0%) |
| Structure data [36. Inclusion of a printed consent form contrary to recommendations (NA if not from hospitals)] | 0 (0%) | 11 (15%) | 62 (85%) |
